# The Ecological Transformation of Successful Intelligence: How High-Stakes Professional Contexts Reshape the Functional Architecture of the Triarchic Model

**DOI:** 10.3390/jintelligence14060102

**Published:** 2026-06-08

**Authors:** Yang Yu, Yinchun Wang, Liye Xie, Yongkang Wu

**Affiliations:** School of Foreign Languages, Dalian Maritime University, Dalian 116026, China; wangyinchun@dlmu.edu.cn (Y.W.); xieliye@dlmu.edu.cn (L.X.); kk191@dlmu.edu.cn (Y.W.)

**Keywords:** triarchic theory of intelligence, ecological context, high-stakes professional cognition, distributed cognition, context-dependent intelligence

## Abstract

This conceptual integrative review and theoretical proposal investigates how the functional architecture of Sternberg’s Triarchic Theory of Intelligence is reconfigured when the framework is translocated from low-risk academic settings, in which analytical intelligence predominates, to high-stakes professional environments characterised by extreme cognitive load, temporal compression, irreversible consequences, and multicultural team dynamics. To construct a mechanistic account of this translocation, we integrate the triarchic framework with three complementary cognitive–ecological traditions—Cognitive Load Theory, the three-level model of Situational Awareness, and the distributed-cognition tradition—and we use the maritime industry as a paradigmatic case where communication failures are directly implicated in catastrophic outcomes. On this basis we propose a Context-Dependent Reweighting Model of Successful Intelligence which maps how, under high-stakes conditions, practical intelligence shifts from a supporting role to a central, integrative function that operates in part through distributed cognitive systems, while creative intelligence assumes elevated weight for adaptive problem-solving when standardised procedures fail. We treat this reweighting as a theoretical proposition supported by convergent but heterogeneous secondary evidence, and we frame the cross-domain extension to aviation, emergency medicine, military operations, and other safety-critical sectors as theoretically plausible parallels and hypotheses for future empirical testing rather than as established empirical claims. The review concludes by articulating implications for intelligence research, proposing a pedagogical framework operationalised through a Triarchic Maritime ESP curriculum, and explicitly delimiting the boundary conditions and limitations of the present contribution.

## 1. Introduction

### 1.1. The Context-Dependency Problem in Successful Intelligence Theory

A foundational claim of Sternberg’s (1985, 1997) Triarchic Theory of Intelligence is that successful functioning in any environment depends not on a single cognitive capacity but on the dynamic interplay of analytical, creative, and practical abilities. This triarchic architecture has proven remarkably productive as a heuristic for understanding human competence, generating a substantial body of research across educational psychology, organisational behaviour, and applied linguistics (Sternberg, 2021; Sternberg et al., 2000). Yet the theory contains a deeper theoretical proposition that has received comparatively little systematic attention: the claim that intelligence is fundamentally context-dependent—that what constitutes “intelligent” behaviour is shaped by the specific ecological demands of the environment in which an individual operates (Sternberg, 1999).

This context-dependency claim carries a profound but largely untested implication. If the ecological demands of different environments are qualitatively distinct, then the functional relationships among the three intelligences—their relative weighting, their modes of interaction, and their operational dynamics—should also vary systematically across contexts. The vast majority of empirical and theoretical work on successful intelligence has been conducted within a remarkably narrow ecological band: formal educational settings, particularly academic contexts in higher-education institutions (Sternberg, 2011; Yu et al., 2025). The ecological validity of intelligence constructs has been recognised as a significant concern across cognitive science (Kihlstrom, 2021; Vaughan & Birney, 2024), and the restriction of successful-intelligence research to academic environments represents a particular instance of this broader limitation.

### 1.2. High-Stakes Professional Environments as a Theoretical Testing Ground

The theoretical gap identified above can be addressed by examining environments that occupy the opposite end of the ecological spectrum from formal education. High-stakes professional environments—defined here as operational contexts where cognitive processing occurs under extreme temporal compression, where information is incomplete and rapidly evolving, where errors carry severe and often irreversible consequences, and where effective performance requires real-time coordination among culturally diverse team members—provide a natural testing ground for investigating the context-dependency of successful intelligence.

Such environments share a cluster of defining characteristics that distinguish them qualitatively from academic settings: temporal compression, asymmetric and severe consequence structure, volatile information ecology, and multicultural and hierarchically complex social ecology. These characteristics constitute the shared ecological signature of professional environments as diverse as maritime navigation, aviation, emergency medicine, military operations, and surgical teamwork (Endsley, 1995; Hutchins, 1995a; Kahneman, 2011; Klein et al., 2018). Cognitive-ergonomic research on these settings (Hollan et al., 2000; Pelgrim et al., 2023; Hissink et al., 2025) has further documented that professionals in these environments require not merely routine competence but the capacity to respond effectively to novel and changing conditions—a capacity that maps directly onto the coordinated deployment of creative and practical intelligence within the triarchic framework.

### 1.3. The Maritime Domain as the Central Paradigmatic Case

To investigate this ecological transformation of successful intelligence, the present review takes the maritime industry as the central paradigmatic case. Our selection of maritime—over the equally legitimate candidacies of aviation, military operations, and emergency medicine—rests on six substantive grounds rather than on convenience.

First, the maritime domain uniquely concentrates all four defining ecological features of high-stakes professional environments—extreme temporal compression at decisive moments, asymmetric and severe consequence structure, volatile information ecology, and multicultural social ecology—within a single operational setting. Aviation, by contrast, is generally less linguistically and culturally heterogeneous at the cockpit level, where cockpits are typically staffed by co-nationals or by speakers of a common operational English; military operations vary widely in their ecological signature depending on service branch and mission type; and emergency medicine is linguistically and culturally diverse but operates within institutional rather than globally distributed settings. Second, maritime work has been the subject of foundational ethnographic research in cognitive science—most notably Hutchins’s (1995a) Cognition in the Wild, an ethnographic study of navigational work aboard the U.S. Navy ship USS Palau—that provides an evidentiary base unmatched in other high-stakes domains. Third, the regulatory framework governing maritime communication (the IMO’s STCW Convention and the SMCP) is unusually well-codified and globally standardised, providing a stable referent against which the operation of the three intelligences can be analysed. Fourth, the multicultural composition of modern ship crews—typically drawn from a wide spectrum of national, linguistic, and cultural backgrounds operating in extended isolation aboard the same vessel—creates an unusually intense and analytically tractable setting for investigating cross-cultural practical intelligence. Fifth, the maritime industry has been the subject of an unusually rich and rapidly growing recent empirical literature on cognitive workload, situational awareness, communication failure, and team dynamics. Sixth, the maritime industry generates an unusually high public-safety and environmental burden when communication and cognition fail, lending the analysis substantive practical relevance beyond its theoretical contribution.

The bridge of a modern commercial vessel constitutes a cognitively extreme sociotechnical system in which officers must simultaneously process information from multiple technological sources—radar, ECDIS, AIS, and VHF radio communications—while making real-time navigational decisions under severe time pressure (Parisi & Nathanael, 2026; Petermann & Alsos, 2026). English serves as the mandated working language of international shipping, formalised through the IMO’s Standard Marine Communication Phrases (SMCP) and the STCW Convention requirements. Studies analysing maritime casualty data have consistently found that human factors contribute to the majority of maritime incidents, with miscommunication, inadequate situational awareness, and failures in crew coordination emerging as recurrent causal themes (Aslan et al., 2024; Cicek et al., 2025; Khan et al., 2026; Liu et al., 2025).

The theoretical insights derived from the maritime analysis are, however, not intended to remain domain-specific. The insights are, we will argue, systematically transferable to other high-stakes professional domains—although such transfers must be framed as theoretically plausible parallels and as hypotheses for future empirical testing, not as established empirical findings.

### 1.4. Purpose, Research Questions, and Theoretical Contributions

The principal aim of this conceptual integrative review and theoretical proposal is to advance intelligence theory by characterising, at a mechanistic level, how the functional architecture of successful intelligence is reconfigured under the ecological conditions of high-stakes professional environments—and, on that basis, to propose a pedagogical framework that operationalises this reconfigured architecture for professional training. We pursue this aim through three numbered objectives, each linked to a specific research question.

Objective 1 (theoretical integration). We construct an integrated theoretical framework by augmenting Sternberg’s triarchic architecture with three complementary cognitive–ecological traditions: Cognitive Load Theory (Sweller, 1988, 2011), Endsley’s (1995) three-level model of Situational Awareness, and the distributed-cognition tradition initiated by (Hutchins, 1995a, 1995b; Hollan et al., 2000). This integration addresses Research Question 1: What integrated theoretical apparatus is required to characterise successful intelligence as it operates in high-stakes professional environments?

Objective 2 (case analysis). Using the maritime domain as the central paradigmatic case, we conduct an integrative analysis that maps the triarchic intelligences onto the specific cognitive and communicative demands of high-stakes professional operations. This analysis addresses Research Question 2: How is the functional architecture of successful intelligence—the relative weighting and interaction of analytical, creative, and practical intelligence—reconfigured under high-stakes ecological conditions?

Objective 3 (pedagogical translation). We propose a Context-Dependent Reweighting Model and derive from it a set of pedagogical design principles for cultivating holistic professional competence in high-stakes domains. These principles are illustrated through a Maritime English for Specific Purposes (ESP) framework but are articulated at a level of generality that supports transfer to other high-consequence professional training contexts. This addresses Research Question 3: What pedagogical principles follow from the proposed reweighting, and how can they be operationalised in a domain-specific curriculum architecture?

### 1.5. Methodological Approach and Scope of the Review

This section specifies the methodological approach by which the present contribution was developed. The work is best characterised, following the terminology recommended by Torraco (2016) and adopted in recent contributions to the conceptual-review literature, as a conceptual integrative review and theoretical proposal. This designation has three implications that we wish to make explicit. First, the aim of the work is theoretical synthesis and the advancement of conceptual understanding, not effect-size synthesis or the production of new primary data. Second, the present manuscript is not a PRISMA-style systematic review (which would require a different research question, typically focused on quantitative outcomes), although the present work follows PRISMA-informed reporting standards for transparency. Third, the evidentiary base of the review is secondary—comprising published theoretical, empirical, and ethnographic work by other researchers—and the analytical contribution lies in the integration of that base under a novel theoretical synthesis.

**Database coverage.** The literature search drew on five sources: Web of Science Core Collection (SSCI, SCIE, A&HCI, ESCI), Scopus, PsycINFO, ERIC, and the International Maritime Organization’s publication archive. Web of Science Core Collection and Scopus were used for the principal evidence base; PsycINFO was used to ensure full coverage of the intelligence-research literature; ERIC was used to ensure full coverage of the language-pedagogy and ESP literature; and the IMO archive was used for the regulatory and operational documentation specific to the maritime case.

**Date range.** The principal empirical evidence base comprises sources published between 2018 and 2026, selected to ensure currency, with the maritime, cognitive-load, and situational-awareness studies over-represented in the more recent end of the range (2024–2026) to reflect rapid recent development in these areas. Foundational theoretical sources from before 2018 were retained when they constituted the original statement of the theory in question—Sternberg (1985, 1997, 1999) for the triarchic framework, Sweller (1988) for cognitive load, Endsley (1995) for situational awareness, Hutchins (1995a, 1995b) and Hollan et al. (2000) for distributed cognition, Wagner and Sternberg (1985) for tacit knowledge, and Helmreich and Merritt (1998) for the foundational CRM-and-culture literature.

**Search-term clusters.** Seven term clusters were used: (i) “triarchic intelligence” OR “successful intelligence” OR “Sternberg AND intelligence”; (ii) “cognitive load” AND (“theory” OR “instructional design”); (iii) “situational awareness” AND (“maritime” OR “aviation” OR “operational”); (iv) “distributed cognition” OR “cognition in the wild”; (v) “maritime English” OR “maritime communication” OR “SMCP”; (vi) “high-stakes” AND (“communication” OR “cognition” OR “decision-making”); (vii) “tacit knowledge” AND (“professional” OR “expert”).

**Inclusion criteria.** Sources were included if they (a) appeared in peer-reviewed journals, edited scholarly volumes, or scholarly monographs; (b) addressed cognition, communication, intelligence, or expert performance in either academic or high-stakes professional contexts; (c) were written in English or translated into English; and (d) contributed either theoretical or empirical material directly relevant to the research questions specified in Section 1.4.

**Exclusion criteria.** Sources were excluded if they (a) appeared only in grey literature or in venues without peer review (with the exception of IMO regulatory documents, which constitute primary regulatory sources); (b) addressed cognition or communication only at the neural or sub-personal level without analytical purchase on the constructs of interest; or (c) duplicated earlier findings without offering substantive theoretical or empirical advance.

**Analytical approach.** The evidence base was synthesised into the theoretical framework through a five-stage process: (i) mapping each retrieved source onto the relevant research question; (ii) identifying convergent and divergent findings within each theoretical pillar (triarchic, cognitive load, situational awareness, distributed cognition); (iii) constructing the Context-Dependent Reweighting Model as a theoretical proposition that integrates the convergent findings; (iv) testing the reweighting model against the maritime case as a paradigmatic instance; and (v) extrapolating tentatively to other high-stakes domains, with explicit hedging of cross-domain claims.

**Epistemic markers.** Throughout the manuscript, we adopt a system of explicit epistemic markers to distinguish empirically supported claims (introduced with phrases such as “empirical research has demonstrated…”) from theoretical propositions (introduced with phrases such as “we propose, as a theoretical conjecture…”) and from cross-domain extrapolations (introduced with phrases such as “by extrapolation to other high-stakes domains, we hypothesise…”). This convention is intended to permit the reader to evaluate the epistemic status of each claim independently.

## 2. Theoretical Foundations

Section 2 establishes the integrated theoretical apparatus that underwrites the Context-Dependent Reweighting Model. We begin in Section 2.1 with explicit definitions of the principal constructs deployed throughout the review, and then treat the four constitutive theoretical pillars in turn: the triarchic framework in Section 2.2, the distributed-cognition tradition initiated by Hutchins in Section 2.3, Cognitive Load Theory in Section 2.4, and the three-level model of Situational Awareness in Section 2.5. We then offer a critical synthesis in Section 2.6, making explicit the limitations of each constituent theory.

### 2.1. Conceptual Definitions

Before proceeding, we make explicit the operational definitions of the principal constructs deployed in this review. The list in Table 1 is not a glossary of every term used in the manuscript but a focused statement of the constructs that do analytical work in the reweighting model. Where a construct has been defined differently in different traditions, we record the working definition adopted here and indicate the source on which we draw.

### 2.2. Revisiting the Triarchic Theory: From Academic to Professional Contexts

The triarchic theory of intelligence provides the overarching conceptual architecture for both this review and its predecessor. As detailed extensively in Yu et al. (2025), the theory identifies three interdependent forms of intelligence: analytical intelligence, which encompasses the ability to analyse, evaluate, compare, and contrast information; creative intelligence, which involves the capacity to generate novel solutions, cope with unfamiliar situations, and automate newly acquired cognitive processes; and practical intelligence, which enables individuals to adapt to, shape, and select real-world environments to achieve their goals. Rather than recapitulating the full theoretical exposition provided in that earlier work, this section focuses on the dimensions of the theory that acquire heightened significance when the framework is translocated from academic to high-stakes professional contexts.

The construct of tacit knowledge occupies a pivotal position in this translocation. Within the triarchic framework, practical intelligence is operationalised primarily through tacit knowledge, defined as procedural knowledge that is acquired through experience rather than explicit instruction, that is relevant to the attainment of personally valued goals, and that is typically not articulated or directly taught (Wagner & Sternberg, 1985; Sternberg et al., 2000). In academic environments, tacit knowledge manifests as an understanding of unstated departmental expectations, awareness of how to navigate the hidden curriculum, and sensitivity to the pragmatic norms governing interactions with supervisors and peers. While important, the stakes attached to academic tacit knowledge are moderate, and its acquisition can proceed gradually over the course of a degree programme.

In maritime professional contexts, tacit knowledge assumes a qualitatively different character. It encompasses the experienced officer’s ability to sense that a developing traffic situation is deviating from routine patterns before any formal risk indicator has been triggered, the veteran chief engineer’s capacity to detect subtle anomalies in engine-room sounds that signal impending mechanical failure, and the skilled bridge team leader’s intuitive calibration of communication directness based on the cultural backgrounds and competence levels of team members. This form of knowledge is domain-specific, deeply situated, and resistant to codification (Sternberg, 2025b). Its acquisition depends heavily on prolonged immersion in authentic professional contexts, a condition that creates a fundamental pedagogical challenge for maritime education institutions that must prepare students for high-stakes performance before they have accumulated significant sea-going experience.

These functional shifts in the deployment of tacit and creative knowledge align with the conceptual evolution of the triarchic model toward a broader theory of Adaptive Intelligence (Sternberg, 2021). Viewed through Sternberg’s recent PTSI (Person × Task × Situation Interaction) framework (Sternberg, 2025a), the deployment of creative and practical intelligence at sea is not merely a static cognitive capacity, but a highly dynamic interaction shaped by personal attributes, task complexity, and acute situational stressors (e.g., fatigue, multicultural crew composition, and unfolding emergencies). Furthermore, Sternberg’s conceptualisation of intelligence as performance rather than mere competence (Sternberg, 2025b) underscores the critical gap between an individual’s potential under optimal conditions and their actual output under the pressures of real-world practice.

### 2.3. The Distributed-Cognition Tradition: Hutchins and Cognition in the Wild

A central limitation of the original triarchic framework, when applied to high-stakes professional environments, is its methodological individualism: the three intelligences are conceived as properties of a single mind operating on a stable environment. This is an appropriate idealisation for the laboratory and the classroom, but it is severely incomplete as a model of cognition in the bridges, cockpits, and operating rooms in which expert professional work actually unfolds. The distributed-cognition tradition initiated by Hutchins (1995a, 1995b; Hollan et al., 2000) offers the conceptual resources needed to extend the triarchic architecture beyond this individualist idealisation, and its integration into the framework is the principal theoretical innovation of the present review.

Hutchins’s (1995a) Cognition in the Wild is foundational because it was conducted in the very domain that constitutes the paradigmatic case of the present review. Drawing on an extended cognitive ethnography of navigational work aboard the U.S. Navy amphibious-assault ship USS Palau, Hutchins demonstrated that the fix-cycle by which a vessel’s position is determined and propagated through the bridge team is not the product of any single navigator’s cognition. It is the product of a distributed cognitive system in which six or seven crew members, several physical instruments (alidades, gyrocompass repeaters, fathometers, plotting boards, charts, log books), and a regulated sequence of communicative acts together compute and maintain a running representation of the ship’s location relative to a network of hazards. The cognitive achievement—accurate navigation in restricted waters—is achieved not in any one head but across the system as a whole.

Hutchins’s (1995b) companion analysis of the cockpit memory system—in which the speeds for various phases of an aircraft’s approach to landing are not remembered by any individual pilot but are inscribed on speed bugs, called out, and re-inscribed across the cockpit’s representational surfaces—extends the same point into a second high-stakes domain. The subsequent programmatic statement of distributed-cognition theory (Hollan et al., 2000) consolidated the empirical findings into a general framework: cognitive processes can be distributed across the members of a social group, coordinated between internal and external (material or environmental) structure, and distributed through time so that the products of earlier events transform the nature of later events.

The integration of this tradition with the triarchic framework has several specific implications for the present analysis. First, what appears at the level of the individual officer as the operation of practical intelligence is, at the level of the bridge team, the operation of a distributed cognitive system; the officer’s tacit knowledge consists in part of knowing how to participate in this system. Second, the schema-driven processing of expert navigators (Section 2.4) is supported not only by internal cognitive structures but by the external structure of charts, displays, and standardised communicative routines that materialise the schema in the environment. Third, and most importantly for the reweighting model, the reason practical intelligence becomes integrative under high-stakes conditions is precisely that it is the form of intelligence most directly attuned to the operation of distributed cognitive systems: it is the capacity to read, interpret, and coordinate within the socio-technical environment, rather than to manipulate representations in isolation. We make use of this insight throughout Section 3, Section 4 and Section 6.

### 2.4. Cognitive Load Theory: The Constraints on Information Processing in High-Stakes Environments

The third theoretical pillar introduced in this review is cognitive load theory, originally developed by Sweller (1988, 2011) and subsequently refined through extensive empirical research in educational psychology. Cognitive load theory provides a principled account of the limitations of human working memory and their implications for instructional design, an account that is indispensable for understanding how intelligence operates under the extreme information-processing demands characteristic of maritime operations.

The theory distinguishes three types of cognitive load. Intrinsic cognitive load refers to the inherent complexity of the material being learned or the task being performed, determined by the number of interacting elements that must be processed simultaneously in working memory. Extraneous cognitive load arises from the manner in which information is presented or organised, representing cognitive effort that does not contribute to learning or task performance. Germane cognitive load refers to the cognitive effort devoted to the construction and automation of schemas, the organised knowledge structures that enable efficient processing of complex information (Sweller, 2011). Recent work has further explored the parallels between human cognitive load constraints and information processing limitations in artificial intelligence systems (P. Wang et al., 2026), underscoring the universal nature of bounded processing capacity across both biological and computational agents.

In maritime operational contexts, all three forms of cognitive load converge at levels that far exceed those typically encountered in academic settings. The intrinsic cognitive load of bridge watchkeeping, for example, is extraordinarily high because it requires the simultaneous integration of multiple information streams: visual observation of the surrounding sea area, radar returns showing traffic patterns, ECDIS displays depicting the vessel’s position relative to navigational hazards, AIS data providing identity and movement parameters of nearby vessels, VHF radio communications from port authorities and other ships, and internal communication from the engine room and other departments (Jiang & Fan, 2026; Ma et al., 2026). From a distributed-cognition standpoint (Hutchins, 1995a; Hollan et al., 2000), this load is borne not by the officer alone but by the bridge team as a system; expert performance consists in part of offloading processing onto instruments, charts, and the standardised utterances of other team members.

Extraneous cognitive load in maritime settings is generated by factors such as poorly designed bridge instrumentation layouts that require frequent visual transitions between spatially separated displays (Petermann & Alsos, 2026), non-standardised communication procedures across different flag states and shipping companies, and the cognitive overhead of processing English-language communications for non-native speakers who must simultaneously translate, interpret, and respond under time pressure. Research on seafarer mental workload has demonstrated that even experienced navigators show significant increases in cognitive load during complex traffic situations and restricted waterway passages (Deng et al., 2025; Guan et al., 2025; Jiang & Fan, 2026), with measurable neurophysiological indicators including changes in EEG patterns and elevated error rates.

Germane cognitive load, in the context of maritime expertise development, corresponds to the construction of the rich, interconnected schemas that enable expert navigators to perceive meaningful patterns in what would appear to novices as an overwhelming array of disconnected data points. The development of these schemas is precisely what distinguishes expert maritime performance from novice performance, and it is the process that maritime education must facilitate. However, the facilitation of schema construction is critically dependent on managing intrinsic and extraneous load so that sufficient working memory resources remain available for germane processing. When the combined intrinsic and extraneous load exceeds working memory capacity, schema construction ceases and performance degrades, a condition that has direct safety implications in operational contexts (Molloy et al., 2026).

The relevance of cognitive load theory to the triarchic framework is twofold. First, it provides a mechanistic explanation for why practical intelligence and tacit knowledge are so crucial in high-stakes environments. Expert schemas, built through extensive experience, effectively reduce the intrinsic cognitive load of complex tasks by enabling the chunking of multiple information elements into single, integrated knowledge units. An experienced officer does not process radar range, bearing, course, and speed as four separate data points for each contact; these elements are integrated into a single, meaningful gestalt of the traffic situation. This schema-driven processing frees working memory capacity for the higher-order cognitive activities of comprehension, projection, and creative problem-solving. Second, cognitive load theory provides a principled basis for instructional design in maritime ESP courses, guiding decisions about task sequencing, information presentation, and scaffolding strategies that optimise the balance between challenge and support. Research in aviation (Bonyad et al., 2026; Molloy et al., 2026), surgical-team performance, and air traffic control (Gyles & Bearman, 2026) has documented strikingly similar patterns of cognitive overload in high-stakes operational contexts.

### 2.5. Situational Awareness: A Bridge Between Cognition and Action

The fourth theoretical component is Endsley’s (1995) three-level model of situational awareness, which has become the dominant framework for understanding how operators in complex, dynamic systems perceive, comprehend, and anticipate the state of their operational environment. Situational awareness has been extensively studied in aviation and, more recently, in maritime contexts (Adams & Hagl, 2025; Hansen & Nazir, 2024; Parisi & Nathanael, 2026), and its integration with the triarchic theory produces a powerful analytical lens for understanding the cognitive demands of maritime communication.

Endsley’s model defines three hierarchically organised levels of situational awareness. Level 1, perception, involves the detection and recognition of relevant elements in the environment. Level 2, comprehension, involves the integration of perceived elements into a meaningful understanding of the current situation, including an appreciation of their significance relative to one’s goals. Level 3, projection, involves the ability to anticipate the future states of the system based on current understanding, enabling proactive rather than reactive decision-making.

The mapping between these three levels and the triarchic intelligences reveals a structured correspondence that illuminates the cognitive architecture of maritime professional competence. Level 1 situational awareness—the perception of environmental elements—draws primarily on analytical intelligence. The ability to accurately decode a VHF radio message using Standard Marine Communication Phrases, to correctly read a radar display, or to identify a navigational light configuration requires the precise analytical processing of incoming information against stored knowledge structures. Level 2 situational awareness—the comprehension of the situation’s meaning—requires the collaborative operation of analytical and practical intelligence; this is where tacit knowledge begins to play a decisive role. Level 3 situational awareness—the projection of future system states—is the level at which creative intelligence becomes most critically engaged.

The integration of situational awareness theory with the triarchic framework also benefits from complementary insights offered by related decision-making theories. Klein’s recognition-primed decision model describes how experts in time-pressured, high-stakes environments make decisions not through deliberate comparison of options but through the rapid recognition of situational patterns that activate practised courses of action (Abraham et al., 2025; Gyles & Bearman, 2026; Klein et al., 2018). Kahneman’s (2011) dual-process theory further enriches this picture by distinguishing between fast, intuitive System 1 processing and slow, deliberative System 2 processing. In maritime operations, the experienced officer operates predominantly through System 1 during routine watchkeeping, shifting to System 2 when novel or ambiguous situations are detected. Table 2 summarises how Endsley’s three levels of situational awareness map onto the triarchic intelligences in maritime communication contexts.

### 2.6. Critical Synthesis: Limitations of the Constituent Theories

Before deploying the integrated framework, we make explicit the limitations of each of the four theoretical pillars on which it rests. Doing so is essential because the integration we propose is not an uncritical aggregation; rather, we treat the four theories as mutually compensating, with the strengths of each addressing some of the weaknesses of the others. Identifying those weaknesses in advance prevents over-claiming on the basis of any one of them.

**Limitations of the Triarchic Theory.** The triarchic framework has been criticised on several grounds, including the difficulty of operationalising the three intelligences in psychometrically discriminable ways (Brody, 2003), the relatively modest incremental validity of triarchic measures beyond conventional analytical assessments in some studies, and the framework’s methodological individualism—it treats intelligence as a property of an individual mind rather than of a person-in-context, despite Sternberg’s repeated emphasis on the contextual subtheory. The distributed-cognition integration (Section 2.3) is intended to address this last limitation, and the explicit modelling of expertise-level variation (Section 7.3) is intended to address questions about how the three intelligences mature across the professional life course.

**Limitations of Cognitive Load Theory.** CLT has been criticised for the conceptual instability of the intrinsic/extraneous/germane distinction, which is theoretically motivated but empirically difficult to dissociate; for its reliance on self-report measures of load whose validity has been disputed; and for its origin in instructional rather than operational settings, which raises questions about the direct applicability of laboratory findings to high-tempo professional work. In our treatment, we use CLT primarily as a mechanism story—that schemas reduce intrinsic load and free working memory for higher-order operations—rather than as a source of precise quantitative predictions, and we explicitly hedge where the construct-validity literature is unsettled.

**Limitations of Situational Awareness Theory.** Endsley’s three-level model has been criticised for treating SA as a psychological state that can be measured at an instant rather than as a process unfolding in time; for under-specifying the role of team-level and distributed-system SA relative to individual SA; and for the difficulty of discriminating SA empirically from related constructs such as expertise, comprehension, and metacognition. Our use of the model retains its analytic value as a vocabulary for distinguishing perception, comprehension, and projection, while drawing on the distributed-cognition tradition to address its individualism.

**Limitations of the Distributed-Cognition Tradition.** The distributed-cognition tradition is rich ethnographically but offers fewer easily testable quantitative predictions than CLT or SA, and the boundary of a “cognitive system” has been a matter of long-running dispute. We use Hutchins’s framework principally as a corrective to the methodological individualism of the other three pillars and as the empirical anchor for the maritime case (Section 3), rather than as the basis for quantitative inference.

The integrated framework we deploy in Section 3, Section 4, Section 5 and Section 6 is therefore best understood as a calibrated synthesis in which the limitations of each constituent theory are partially addressed by the strengths of the others. Where empirical claims are advanced, we hedge them; where the synthesis moves beyond what any constituent theory can underwrite on its own, we mark the move explicitly.

## 3. The Maritime Domain as a Paradigmatic High-Stakes Environment

Having established the integrated theoretical framework, this section applies it to a detailed analysis of the maritime domain as a paradigmatic high-stakes professional environment. The analysis is anchored, where appropriate, in Hutchins’s (1995a) foundational ethnography of navigational work aboard the USS Palau, supplemented by the rapidly growing recent empirical literature on cognitive load, situational awareness, and communication in commercial shipping. The analysis is organised around three interconnected dimensions that characterise high-stakes professional environments broadly: operational complexity, crisis communication, and multicultural team dynamics.

### 3.1. Operational Complexity: Multitasking, Information Integration, and Real-Time Decision-Making

The foundational empirical illustration of operational complexity in maritime cognition remains Hutchins’s (1995a) analysis of the fix cycle aboard the USS Palau. In Hutchins’s account, fixing the ship’s position requires the coordinated activity of a bearing-taker on each wing of the bridge, a plotter, a recorder, the conning officer, and a fathometer operator, mediated by alidades, gyrocompass repeaters, plotting charts, sound-powered telephones, and a standardised sequence of verbal exchanges. The cognitive achievement of the bridge—a continually updated representation of the ship’s location, course, and proximity to hazards—is the product of a distributed cognitive system, not of any single navigator’s mental computation. Modern commercial bridges have replaced some of these instruments with electronic equivalents (ECDIS, AIS, integrated bridge systems), but the underlying point—that operational competence consists of skilful participation in a distributed cognitive system—survives the technological transformation intact (Petermann & Alsos, 2026; Tang et al., 2025).

Modern commercial vessels remain complex sociotechnical systems in which navigational safety depends on the continuous integration of information from diverse technological and human sources. The bridge of a large merchant vessel constitutes an information-rich environment where the officer of the watch must simultaneously monitor radar displays, ECDIS, AIS data feeds, meteorological instruments, and visual conditions through the bridge windows, while maintaining VHF radio communications with vessel traffic services, other ships, and port authorities (Cheng et al., 2024; Petermann & Alsos, 2026). Research on seafarer mental workload has documented the substantial cognitive demands imposed by this multitasking environment, with EEG-based studies identifying significant increases in cognitive load during complex traffic situations (Guan et al., 2025; Jiang & Fan, 2026; Ma et al., 2026), and eye-tracking research revealing constrained visual scanning patterns under increasing traffic density (Hung & Chang, 2025; Petrovic & Vujicic, 2025).

The linguistic dimension of this operational complexity is frequently underestimated. Maritime communication is not simply the application of general English proficiency to a maritime context; it involves a specialised register governed by strict conventions, standardised phraseology, and domain-specific pragmatic norms. The IMO’s Standard Marine Communication Phrases represent an attempt to reduce communicative ambiguity through linguistic standardisation, providing prescribed formulations for routine and emergency communications (Zhao & Wei, 2026). However, research on ship-shore radio communication has revealed that even experienced operators frequently deviate from SMCP conventions, employing non-standard formulations, code-switching between languages, and relying on contextual inference to fill communicative gaps (Guo et al., 2026).

From the perspective of the triarchic framework, routine maritime operations place heavy demands on analytical intelligence for the accurate processing of standardised communications and the precise interpretation of instrumental data, while simultaneously requiring practical intelligence for the contextual adaptation of communicative behaviour. Cross-domain parallels—air traffic controllers tracking multiple aircraft while maintaining verbal coordination with pilots, or emergency-department physicians integrating monitor data, laboratory results, and nursing reports while making rapid treatment decisions—are theoretically plausible and we raise them throughout, but we present them as hypotheses for empirical testing rather than as established findings.

### 3.2. Crisis and Emergency Communication: Time Pressure, Incomplete Information, and Emotional Interference

Emergency situations at sea represent the most extreme manifestation of the cognitive demands discussed above. When a vessel encounters a fire, flooding, collision, or grounding, the temporal window for effective decision-making and communication compresses dramatically, the quality of available information degrades, and the emotional and physiological responses of crew members introduce additional sources of cognitive interference (Cinar et al., 2026; Georgiou et al., 2026).

Research on human factors in maritime emergencies has consistently identified communication failure as a critical link in accident causation chains. Analyses of maritime accident investigation reports reveal that communication breakdowns contribute to a substantial proportion of casualties, with miscommunication and misinterpretation together accounting for the majority of observed communication errors (Khan et al., 2026; Liu et al., 2025). These failures are not primarily attributable to deficient vocabulary or grammatical knowledge; rather, they reflect breakdowns in the higher-order cognitive processes of situational comprehension, anticipatory projection, and adaptive communication that become most critical precisely when they are most difficult to sustain.

Research on decision-making under time pressure, drawing on the naturalistic decision-making tradition, has demonstrated that experts in high-stakes domains rely heavily on recognition-primed decision processes rather than analytical comparison of alternatives (Abraham et al., 2025; Gyles & Bearman, 2026). This finding has profound implications for Maritime English pedagogy: the ability to communicate effectively in emergencies depends not on the conscious recall and application of memorised phrases but on the existence of deeply automatised communication schemas that can be activated rapidly under extreme cognitive load. By extrapolation to other high-stakes domains, we hypothesise that an analogous mechanism operates in aviation incidents (Haji Amiri et al., 2025; Terzioğlu, 2024) and in operating-room emergencies addressed through the SBAR framework (Shrivastava et al., 2025); the cross-domain claim is offered as theoretically plausible rather than as empirically established.

### 3.3. Multicultural Crew Dynamics: Cross-Cultural Communication, Power Distance, and Linguistic Diversity

The third dimension of maritime cognitive and communicative complexity arises from the multicultural composition of modern ship crews. The globalisation of the maritime labour market has produced vessels where officers and ratings represent a dozen or more nationalities, each bringing distinct cultural values, communication styles, and expectations regarding authority and interpersonal interaction (Khan et al., 2026; Miskovic & Wang, 2025).

Khan et al. (2026) applied a Bayesian Network framework to 550 maritime accident investigations, using Natural Language Processing to extract indicators including crew nationality composition, language diversity measured through the Shannon Index, cultural diversity assessed through the Hofstede Index, and adherence to Standard Maritime Communication Phrases. Their results demonstrated that high language diversity and low proficiency substantially increase communication failures, with miscommunication and misinterpretation together accounting for sixty-eight percent of observed errors. Critically, their scenario analysis demonstrated that aligning language training, SMCP use, and cultural familiarisation could reduce total loss probability on large container ships from approximately twenty-three percent under stressed human-factor conditions to three percent under best-practice communication settings.

Beyond linguistic proficiency, cultural dimensions exert profound effects on safety-critical communication behaviours. In high power-distance cultures, junior officers may be reluctant to challenge the decisions of senior officers or to report observed errors, even when such challenges or reports are essential for safety (Kuzu, 2026; Yao et al., 2026). The concept of Bridge Resource Management, adapted from aviation’s Crew Resource Management framework, has been introduced into maritime education to address these teamwork and communication challenges (Dang et al., 2025; Lehtimaki & Teperi, 2026).

From the triarchic perspective, multicultural crew communication places exceptional demands on practical intelligence. The ability to adapt one’s communicative style to the cultural expectations of interlocutors from diverse backgrounds, to recognise and manage the effects of cultural differences on team dynamics, and to build sufficient interpersonal trust to enable frank safety-related communication across cultural boundaries represents a sophisticated form of practical intelligence that is deeply dependent on tacit knowledge. Aviation CRM research (Haji Amiri et al., 2025; Helmreich & Merritt, 1998) and military coalition-language research (Siegel et al., 2025) report structurally similar patterns; we treat these convergent observations as theoretically plausible parallels rather than as established cross-domain empirical claims, and we return to the boundary conditions on this extrapolation in Section 7.4.

## 4. Mapping Triarchic Intelligence onto High-Stakes Professional Competencies: Evidence from the Maritime Case

Building on the theoretical foundations established in Section 2 and the contextual review presented in Section 3, this section undertakes the core analytical task of the review: the systematic mapping of the three triarchic intelligences onto the specific competencies required for effective Maritime English communication. Where the earlier review by Yu et al. (2025) operated within the relatively stable communicative ecology of academia, the present analysis confronts the volatile, high-consequence, and temporally compressed communicative ecology of maritime operations. Throughout, we treat the relative weighting of the three intelligences as a theoretical proposition supported by convergent secondary evidence rather than as a quantitatively precise empirical claim.

### 4.1. Analytical Intelligence in Maritime Esp

Analytical intelligence in the maritime ESP context encompasses the cognitive operations required for the accurate perception, decoding, and evaluation of linguistic and informational inputs relevant to navigational safety and operational efficiency. The most prominent manifestation is the precise comprehension and production of Standard Marine Communication Phrases, which requires the analytical capacity to select the correct phrase for a given communicative situation, to parse incoming messages with phonological precision despite the degraded audio quality typical of VHF radio transmission, and to detect deviations from standard formulations (Guo et al., 2026; Zhao & Wei, 2026). Beyond standardised phraseology, analytical intelligence is engaged in the critical interpretation of navigational information presented through multiple modalities including radar, ECDIS, and meteorological displays.

The critical distinction between analytical intelligence in academic and maritime contexts lies in the temporal dimension. In academic settings, analytical processing can proceed at a pace determined by the learner, with opportunities for rereading, reflection, and revision. In maritime operations, analytical processing must frequently occur in real time, under conditions where delayed comprehension may result in collision, grounding, or other catastrophic outcomes. This temporal compression means that the analytical intelligence required for maritime ESP is not merely the same cognitive capacity operating faster; it is a qualitatively different form of analytical processing that depends on the availability of well-developed schemas to support rapid pattern recognition.

### 4.2. Creative Intelligence in Maritime Esp

Creative intelligence in the maritime context manifests most visibly in situations where standardised procedures and routine communication patterns prove insufficient to address the demands of an unfolding situation. The maritime domain generates a continuous stream of situations that, while sharing structural similarities with previously encountered scenarios, present novel configurations of variables that resist purely routine responses. In the specific domain of Maritime English communication, creative intelligence is engaged when officers must reformulate a message that has not been understood by a non-native English-speaking interlocutor, when they must improvise communication strategies following the failure of primary VHF equipment, or when they must construct novel linguistic formulations to describe an unprecedented situation for which no standard phrase exists.

The PTSI theory of creativity (Sternberg, 2025a) provides a useful framework for understanding these demands: the creative response is shaped not only by the individual’s cognitive capacity but by the interaction between the person’s knowledge and skills, the specific characteristics of the communicative task, and the situational context including time pressure, crew composition, and environmental conditions. Research on maritime accident causation provides indirect but compelling evidence for the critical role of creative intelligence in maritime safety (Liu et al., 2025; Ma et al., 2025). Adaptive-expertise research in healthcare (Pelgrim et al., 2023; Hissink et al., 2025) and on strategic improvisation in emergency response (Mamédio et al., 2025) reports structurally analogous patterns, which we treat as theoretically plausible cross-domain parallels rather than as established empirical equivalences.

### 4.3. Practical Intelligence and Tacit Knowledge in Maritime Esp

Practical intelligence, operationalised through tacit knowledge, represents the dimension of the triarchic framework that undergoes the most dramatic amplification when the theory is translocated from academic to maritime professional contexts. The practical intelligence required for effective maritime communication operates across multiple interconnected domains. At the interpersonal level, it includes the ability to calibrate one’s communicative directness to the cultural expectations and hierarchical sensitivities of interlocutors from diverse backgrounds, to detect and respond to non-verbal cues indicating confusion, disagreement, or distress, and to manage one’s own emotional and cognitive state under conditions of extreme stress (Khan et al., 2026; Kuzu, 2026). From a distributed-cognition perspective (Hutchins, 1995a; Hollan et al., 2000), what we have called practical intelligence is also, at the team level, the capacity to participate skilfully in a distributed cognitive system—to read its representational artefacts, to coordinate one’s communicative contributions with those of other team members, and to maintain the system-level coherence on which safe operation depends.

The challenge for maritime ESP pedagogy is therefore not simply to teach practical intelligence as an additional content area but to create learning environments that facilitate the acquisition of tacit knowledge in the absence of the extended professional immersion through which such knowledge is naturally acquired. This challenge is addressed in the pedagogical framework proposed in Section 6, which draws on simulation-based training, scenario-based learning, and structured reflective practice as mechanisms for accelerating the development of practical intelligence. Table 3 summarises how the three triarchic intelligences map onto the key competencies required in Maritime ESP contexts.

## 5. A Diagnostic Case Study: Evaluating Current Domain-Specific Pedagogy Against the Triarchic Framework

Having established the theoretical demand for a balanced development of all three triarchic intelligences in high-stakes professional communication, this section turns a diagnostic lens on the pedagogical approaches currently employed in the specific case of Maritime English education. The purpose is not to provide a comprehensive review of maritime pedagogy per se, but to use this case to illustrate how the analytical bias identified in intelligence-informed education (Yu et al., 2025) manifests in—and is potentially amplified by—the specific ecological demands of high-stakes professional training.

### 5.1. Smcp-Based Instruction and the Analytical Bias

The dominant approach to Maritime English instruction worldwide centres on the teaching and practice of the IMO’s Standard Marine Communication Phrases. This approach, which aligns with the STCW Convention’s requirements for maritime communication competence, typically involves the systematic presentation of SMCP vocabulary and phrase structures, drilling exercises focused on accurate production and comprehension, and assessment through written and oral tests that measure the ability to recall and deploy standard formulations (Guo et al., 2026; Nyairo et al., 2025). From the perspective of the triarchic framework, SMCP-based instruction is overwhelmingly oriented toward the development of analytical intelligence; it provides no systematic mechanism for developing creative intelligence (the ability to adapt and improvise) or practical intelligence (the ability to read contextual cues and manage interpersonal dynamics).

### 5.2. Simulator-Based Training: Potential and Limitations

Maritime simulators represent the most promising existing platform for the development of creative and practical intelligence in maritime education. Full-mission bridge simulators, engine room simulators, and communication simulators can create immersive, dynamic scenarios that approximate the cognitive demands of actual maritime operations (Georgiou et al., 2026; Nyairo et al., 2025; Sellberg & Sharma, 2025). Research on simulator-based maritime training has demonstrated positive effects on teamwork behaviours, situational awareness, and decision-making quality (Chan et al., 2025; Dang et al., 2025), and VR-based studies have shown enhanced learning benefits and engagement among maritime students (Bacnar et al., 2024; Liker et al., 2025). However, the integration of language training into simulator-based exercises remains underdeveloped.

### 5.3. Task-Based and Scenario-Based Approaches

Task-based language teaching has gained increasing attention in ESP pedagogy as a means of creating authentic, purposeful communicative activities that engage learners in meaning-focused interaction (Basturkmen, 2025; Fujita & Shintani, 2025; Yan, 2025). The application of task-based and scenario-based approaches to Maritime English instruction, while growing, remains limited in scope and sophistication. Where such approaches have been implemented, they have typically focused on relatively constrained communicative tasks such as VHF radio exchanges or port entry procedures, without systematically addressing the full range of cognitive demands identified in this review.

The pedagogical gap between what is systematically taught and what professional performance actually demands is, by extrapolation rather than direct empirical demonstration, not unique to Maritime English; parallel analytical biases have been noted in aviation English instruction (A. W. Wang & Friginal, 2025), in medical communication training (Shrivastava et al., 2025), and in military language training (Siegel et al., 2025). The strength of these cross-domain parallels remains an open empirical question (see Section 7.4 for explicit boundary conditions).

## 6. From Theory to Pedagogy: A Triarchic Framework for High-Stakes Professional Communication

The theoretical analysis and diagnostic case study presented in the preceding sections establish both the need for and the principles underlying a new approach to professional communication pedagogy in high-stakes domains. This section presents a pedagogical framework that translates the Context-Dependent Reweighting Model into actionable instructional design principles. The framework is articulated at two levels: first, as a set of general pedagogical transformations that apply across high-stakes professional communication training; and second, as a fully operationalised Maritime English for Specific Purposes (ESP) curriculum, including a 32-week curriculum architecture (Section 6.4) and a competency assessment matrix (Section 6.5), in direct response to the reviewers’ request for concrete operationalisation.

### 6.1. Transformation One: From Low-Risk to High-Risk Contexts Through Cognitive Load Management

The first transformation addresses the shift from the relatively forgiving cognitive environment of academic settings to the high-stakes, high-load environment of maritime operations. The framework adopts a graduated cognitive load approach in which students are initially exposed to maritime communication tasks with reduced element interactivity, allowing them to build foundational schemas without cognitive overload. As proficiency develops, task complexity is systematically increased through the introduction of additional information sources, time pressure, concurrent demands, and unexpected complications.

Concretely, this principle translates into a three-phase task design architecture. In Phase 1 (Foundation), students engage with isolated maritime communication tasks that target analytical intelligence. In Phase 2 (Integration), tasks combine multiple information sources and introduce moderate time pressure, engaging both analytical and practical intelligence. In Phase 3 (Performance), students confront full-complexity scenarios in simulator environments that approximate the cognitive demands of actual maritime operations, with all three intelligences simultaneously engaged.

### 6.2. Transformation Two: From Monocultural to Multicultural Contexts Through Cross-Cultural Practical Intelligence

The second transformation addresses the shift from the relatively homogeneous cultural environment of most academic classrooms to the radically multicultural environment of international shipping. The framework addresses this transformation through the systematic incorporation of cross-cultural communication scenarios into all phases of instruction: in Phase 1, awareness-raising activities introduce students to cultural dimensions most relevant to maritime communication; in Phase 2, structured role-play activities require communication with simulated interlocutors from different cultural backgrounds; in Phase 3, simulator exercises include multicultural crew compositions, requiring students to manage interpersonal dynamics of culturally diverse teams while addressing technical demands of the navigational scenario.

### 6.3. Transformation Three: From Delayed Written Production to Real-Time Oral Communication Under Pressure

The third transformation addresses the shift from the predominantly written, delayed-production communicative mode of academic settings to the predominantly oral, real-time communicative mode of maritime operations. The framework addresses this transformation by progressively shifting the balance of communicative activities from written to oral modes across the three phases. A distinctive feature is the incorporation of communication breakdown and recovery exercises, in which scenarios are deliberately designed to produce communication failures that students must detect, diagnose, and resolve in real time. These exercises directly target creative intelligence by requiring students to generate adaptive communicative solutions when standard approaches fail.

### 6.4. A 32-Week Curriculum Architecture: Operationalising the Framework

To address reviewer requests for explicit pedagogical operationalisation, we specify a 32-week Triarchic Maritime ESP curriculum architecture, designed for delivery across two 16-week semesters at a maritime university and scalable in either direction (a 16-week intensive variant for in-service training, or a four-semester expansion for full-degree maritime English majors). The architecture is summarised in Table 4. Each phase aligns specific instructional activities with target intelligences and with Endsley’s SA levels, and each phase culminates in an assessment moment drawn from the matrix specified in Section 6.5.

Three architectural commitments distinguish the curriculum. First, the weighting of the three intelligences shifts deliberately across the phases: analytical predominance in weeks 1–8, balanced engagement from week 9 to week 20, and creative- and practical-led integration from week 21 to week 31. This shift mirrors, in compressed pedagogical form, the wider transformation theorised in Section 7. Second, each phase culminates in an assessment moment whose construct alignment is specified in Table 5; this permits both formative and summative evidence on each intelligence independently. Third, the curriculum is bookended by an introductory module (weeks 1–4) and a transition-to-practice module (week 32) that explicitly frame the curriculum as a preparation for, not a substitute for, shipboard tacit-knowledge acquisition.

### 6.5. Assessing Triarchic Maritime ESP Competencies

The triarchic framework implies that assessment must evaluate not only analytical competencies but also creative competencies (such as adaptive communication under novel conditions) and practical competencies (such as cross-cultural communicative effectiveness and team coordination). Table 5 specifies the construct, the recommended assessment instrument, the timing, and the validity considerations for each of the three intelligences.

### 6.6. An Illustrative Multi-Phase Task Sequence

To concretise the framework’s pedagogical principles, this section presents an illustrative multi-phase task sequence based on a realistic maritime scenario: a cargo vessel experiencing a main-engine failure while navigating a congested traffic separation scheme.

In Phase 1, students would work with the written text of the relevant SMCPs for reporting engine failure and requesting assistance, analysing the linguistic structure of these formulations, practising their accurate production, and completing comprehension exercises based on recorded examples of similar communications. This phase targets analytical intelligence through precise linguistic processing.

In Phase 2, students would engage in a tabletop exercise in which they role-play the bridge team of the affected vessel. The exercise would introduce a complication: the VTS operator speaks English with a strong accent that requires careful listening and occasional requests for repetition. This phase engages both analytical intelligence through accurate message processing and practical intelligence through the management of communicative difficulty with a non-native English-speaking interlocutor.

In Phase 3, the same scenario would be enacted in a full-mission bridge simulator with a multinational student team. The scenario would include additional complications: a second vessel fails to respond to radio warnings, requiring the team to improvise alternative communication strategies; a junior team member from a high power-distance culture hesitates to report a critical observation to the senior officer; and the time pressure of the developing situation compresses the window for effective communication. This phase engages all three intelligences simultaneously.

## 7. Discussion

### 7.1. Theoretical Implications for Intelligence Research

The analysis presented in this review carries implications that extend beyond pedagogy to the theoretical foundations of intelligence research itself. Three implications merit particular attention.

**Figure 1:**
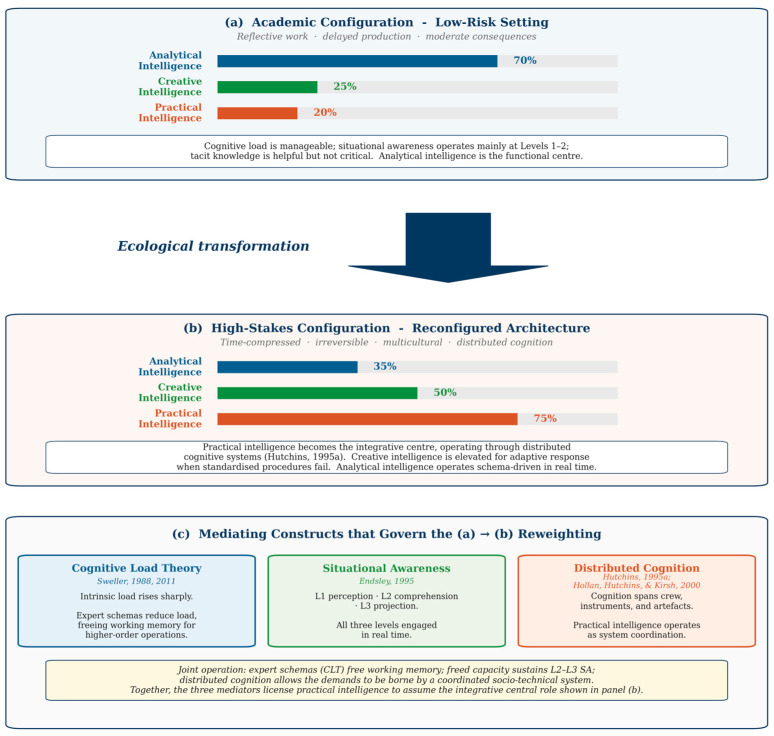
The Context-Dependent Reweighting Model of Successful Intelligence. Panels (**a**,**b**) display the proposed functional reweighting of the three triarchic intelligences as the ecological context shifts from low-risk academic settings to high-stakes professional environments; panel (**c**) specifies the mediating constructs that theoretically govern this reweighting: Cognitive Load Theory (Sweller, 1988, 2011), Situational Awareness (Endsley, 1995), and Distributed Cognition (Hutchins, 1995a; Hollan et al., 2000). The model is advanced as a theoretical proposition supported by convergent secondary evidence, to be tested empirically in future research.

First, the findings support a context-dependent reweighting model of successful intelligence (specified in Figure 1). In academic environments, analytical intelligence occupies the centre of the competence landscape. The present analysis proposes, as a theoretical conjecture supported by convergent secondary evidence, that in high-stakes professional environments this configuration undergoes a fundamental transformation: practical intelligence shifts from the periphery to the centre, while creative intelligence assumes a criticality that it does not possess in academic settings. This reweighting is not a minor quantitative adjustment; it represents a qualitative reconfiguration of the functional architecture of successful intelligence.

Second, the integration of Cognitive Load Theory, Situational Awareness, and the distributed-cognition tradition with the triarchic framework provides a mechanistic account of why this reweighting occurs. Cognitive load theory specifies the mechanism by which expert schemas reduce intrinsic load and free working memory for higher-order operations; the SA model specifies the levels at which the freed capacity is deployed; and the distributed-cognition tradition specifies how the cognitive demands can be borne by a coordinated socio-technical system rather than by a single mind. Practical intelligence is not merely useful in high-stakes environments; on the proposed view, it is a necessary precondition for the effective deployment of both analytical and creative intelligence.

Third, the analysis has implications for the assessment and measurement of intelligence in applied contexts. If the functional architecture of successful intelligence varies systematically with ecological context, then assessment instruments developed for academic settings—which inevitably privilege analytical intelligence—will systematically underestimate the competence of individuals who excel in practical and creative dimensions. The ecological validity of cognitive assessment has been recognised as a significant methodological challenge (Vaughan & Birney, 2024).

### 7.2. Cross-Domain Transferability: Theoretically Plausible Parallels and Hypotheses for Future Testing

In the original submission we presented cross-domain extension as a well-established generalisation. In response to reviewer guidance, we explicitly reframe the cross-domain extension as a set of theoretically plausible parallels and hypotheses for future empirical testing. The structural similarities documented throughout the preceding analysis are real, but the inferential leap from “structural similarity” to “transfer of mechanism” requires direct empirical work in each candidate domain. Table 6 summarises the four principal candidate domains and indicates the strength of the current evidentiary basis for extrapolation.

The asymmetry of evidentiary strength across the four domains is itself theoretically informative: the closer a domain is, in its sociotechnical configuration, to the paradigmatic maritime case, the stronger the warrant for extrapolation. Aviation is the closest cognate; nuclear operations and other safety-critical industries are the most distant. The reweighting model offers a theoretical resource for thinking about each of these domains, but the empirical test of the model in each must be conducted domain by domain.

### 7.3. Variations Across Subcontexts and Expertise Levels

The claim that practical intelligence assumes a central role in high-stakes professional environments requires nuancing in two respects.

**Subcontextual variation within maritime work.** The maritime industry is not internally homogeneous. Routine deep-sea navigation in unrestricted waters places relatively modest demands on creative and practical intelligence relative to congested coastal navigation, ice-covered routes, or emergency-response operations. Pilotage in restricted waterways, by contrast, concentrates exactly the demands theorised in the reweighting model; Hutchins’s (1995a) analysis was conducted in this kind of operational subcontext. The reweighting we propose is sharpest at the high-demand end of the maritime subcontextual spectrum and softer at the low-demand end.

**Expertise-level variation.** The functional architecture of successful intelligence almost certainly varies across the professional career: cadets in their first sea service, third officers in their early bridge watches, senior officers approaching command, and master mariners with decades of experience cannot reasonably be expected to deploy the three intelligences in the same functional configuration. We propose, on the basis of the expertise literature (Klein et al., 2018; Pelgrim et al., 2023), that the reweighting articulated in Figure 1, panel (b) is most fully realised in fully expert professionals; in novice-to-intermediate practitioners, analytical intelligence remains comparatively more prominent because the schemas that would otherwise support tacit, schema-driven processing have not yet been consolidated. This expertise-level qualification has direct pedagogical implications: the graduated cognitive-load architecture specified in Section 6.1 and Section 6.4 is designed precisely to support the trajectory from analytical predominance (novice) toward integrative practical-intelligence centrality (expert).

### 7.4. Boundary Conditions and Limitations of the Present Contribution

We close the discussion with an explicit statement of the boundary conditions and limitations of the present contribution. We identify five.

**First, evidentiary basis.** The Context-Dependent Reweighting Model is advanced as a theoretical proposition supported by convergent but heterogeneous secondary evidence, not as an empirically established finding. The principal evidentiary anchor—Hutchins’s (1995a) ethnography—is qualitatively rich but methodologically ethnographic; the bridge-workload and accident-causation literature on which we draw is more recent and quantitative but does not directly test the reweighting model in the form we propose it. Direct empirical testing of the model is a priority for future research.

**Second, the case-anchored character of the analysis.** The maritime case is treated as paradigmatic, not as representative in any statistical sense. While Section 1.3 motivates the choice on six substantive grounds, the analysis derives its insights from a single (if internally varied) high-stakes domain. The reweighting model may need adjustment in domains whose ecological signature differs systematically from the maritime case (see Section 7.2 and Table 6).

**Third, the cross-domain extrapolation.** As specified in Section 7.2, the extension of the model to aviation, emergency medicine, military coalition operations, and other safety-critical sectors is presented as theoretically plausible and as a hypothesis for future empirical testing, not as an established empirical generalisation. The asymmetric strength of the evidentiary basis across these domains (Table 6) should be borne in mind whenever the model is applied beyond the maritime case.

**Fourth, the limitations of the constituent theories.** Section 2.6 has already specified the limitations of each of the four theoretical pillars on which the reweighting model rests—the construct-validity disputes around the triarchic framework, the difficulty of empirically dissociating the three CLT load types, the SA model’s individualism, and the ethnographic rather than predictive character of distributed-cognition theory. The integrated framework is therefore best understood as a calibrated synthesis, not as a fully validated empirical apparatus.

**Fifth, pedagogical operationalisation.** The 32-week curriculum architecture (Section 6.4) and the competency assessment matrix (Section 6.5) are illustrative operationalisations, not tested implementations. Their efficacy and scalability remain to be established through randomised or quasi-experimental intervention studies with appropriate comparison conditions, and through psychometric work on the assessment instruments specified in Table 5.

### 7.5. Implementation Challenges

While the following challenges are discussed in the context of the maritime domain, they represent general implementation barriers that any attempt to operationalise a triarchic approach to high-stakes professional communication training would need to address. The most significant challenge is the requirement for ESP instructors who possess not only linguistic and pedagogical expertise but also sufficient understanding of the professional domain to design and facilitate the integrated, scenario-based activities the framework demands (Huang & Trent, 2026; H. Wang & Xu, 2026). A second concerns assessment: developing valid, reliable, and practical instruments for higher-order competencies remains a significant research and development challenge (Hsu et al., 2026). A third concerns the resource requirements of simulator-based training, which remain uneven across institutions worldwide (Baradziej et al., 2026; Kitada & Hiwatashi, 2024), although the growing availability of VR-based systems offers a promising pathway (Christopoulos & Stylios, 2024; Muczynski et al., 2025).

## 8. Conclusions

This review has investigated a fundamental but underexplored question in intelligence research: how does the functional architecture of successful intelligence transform when the ecological context shifts from low-risk academic environments to high-stakes professional domains? Using the maritime industry as a paradigmatic case and integrating Sternberg’s triarchic theory with Cognitive Load Theory, Situational Awareness, and the distributed-cognition tradition initiated by Hutchins (1995a), the analysis has produced the following synthetic set of findings.

(1) Context-dependent reweighting is a substantive theoretical proposition, not merely a descriptive observation: the relative functional weighting of analytical, creative, and practical intelligence shifts systematically across qualitatively different ecological contexts (Figure 1).
(2) Practical intelligence, operationalised through tacit knowledge and through skilful participation in distributed cognitive systems, moves from a supporting role in low-risk academic settings to an integrative central role in high-stakes professional environments.
(3) Creative intelligence assumes elevated functional weight under high-stakes conditions because standardised procedures, while necessary, are insufficient to handle the novel configurations of variables that high-stakes operations routinely generate.
(4) Cognitive Load Theory specifies the mechanism—expert schemas reduce intrinsic load and free working memory for higher-order operations—that underwrites the proposed reweighting.
(5) Endsley’s three-level model of Situational Awareness specifies the levels (perception, comprehension, projection) at which the freed working-memory capacity is deployed; the three intelligences map onto these levels in a structured way (Table 2).
(6) The distributed-cognition tradition (Hutchins, 1995a; Hollan et al., 2000) corrects the methodological individualism of the other three pillars by locating cognitive work in the coordinated activity of people, artefacts, and representations across a sociotechnical system.
(7) The pedagogical implications of the reweighting model can be operationalised concretely, as illustrated by the 32-week Triarchic Maritime ESP curriculum (Table 4) and the competency assessment matrix (Table 5) specified in Section 6.
(8) Cross-domain extension to aviation, emergency medicine, military coalition operations, and other safety-critical sectors is theoretically plausible but remains a set of hypotheses for empirical testing (Table 6), not a set of established generalisations.

**Relevance for intelligence research.** The eight findings above support a single overall claim with which we close. If the functional architecture of successful intelligence is indeed ecologically context-dependent in the manner we propose, then the study of intelligence cannot remain epistemically anchored to the educational settings in which it has predominantly been conducted. Ecological context should be recognised as a primary variable in the study of intelligence—not as a background condition to be controlled away, but as a constitutive determinant of what “successful” intelligent functioning consists of. The maritime case is one paradigmatic instance of this larger argument; the intellectual programme it invites is the systematic ecological broadening of intelligence research itself.

We close with the note that the framework proposed here is conceptual; its efficacy and scalability can only be determined through rigorous empirical research. Key priorities include longitudinal intervention studies comparing triarchic-informed instruction with conventional approaches, the development and validation of ecologically valid assessment instruments for creative and practical intelligence in professional contexts, and cross-domain implementation research investigating the framework’s transferability under the boundary conditions specified in Section 7.

## Figures and Tables

**Table 1:** Conceptual definitions of the principal constructs.

Construct	Working Definition Adopted in This Review
**Ecological transformation**	The systematic reconfiguration of the functional architecture of successful intelligence as the operating context shifts along a continuum from low-stakes, reflective settings to high-stakes, time-compressed, irreversible-consequence settings (after Sternberg, 1999; Kihlstrom, 2021).
**High-stakes cognition**	Cognition occurring under the joint pressure of temporal compression, irreversible-consequence asymmetry, volatile information ecology, and multicultural-team coordination demands (Endsley, 1995; Klein et al., 2018).
**Tacit knowledge**	Procedural knowledge that is acquired through experience rather than explicit instruction, that is relevant to the attainment of personally or professionally valued goals, and that is typically not articulated or directly taught (Wagner & Sternberg, 1985; Sternberg et al., 2000).
**Practical intelligence**	The ability to adapt to, shape, and select real-world environments to achieve valued goals, operationalised principally through tacit knowledge and contextual judgement (Sternberg, 1997; Sternberg et al., 2000).
**Adaptive communication**	The capacity to reformulate, repair, and improvise communicative behaviour under operational pressure when standardised protocols prove insufficient—a construct that depends jointly on creative and practical intelligence.
**Contextual reweighting**	The systematic shift in the relative functional weighting of the three triarchic intelligences across qualitatively different ecological contexts; the central theoretical proposition advanced by the present review.
**Distributed cognition**	A theoretical orientation that locates cognitive processes not solely in the individual mind but in the coordinated activity of people, artefacts, and representations across a sociotechnical system (Hutchins, 1995a; Hollan et al., 2000).
**Cognitive ecology**	The set of environmental, technological, social, and temporal features of a context that jointly shape the cognitive demands placed on operators within it (Hutchins, 2010).
**Schema-driven processing**	Information processing supported by organised knowledge structures (schemas) that permit rapid pattern recognition and efficient chunking of complex inputs, thereby reducing intrinsic cognitive load (Sweller, 2011).

**Table 2:** Mapping Endsley’s Situational Awareness Levels to Triarchic Intelligences.

SA Level	Primary Intelligence Engaged	Maritime Communication Example	Cognitive Load Characteristic
Level 1: Perception	Analytical Intelligence	Accurately decoding a VHF radio message using SMCP; precise phonological and lexical processing.	Demands precise analytical processing of incoming information against stored knowledge structures.
Level 2: Comprehension	Analytical + Practical Intelligence	Interpreting the pragmatic force of a port authority’s seemingly routine instruction based on contextual knowledge.	Relies on tacit knowledge and expert schemas to comprehend meaning, reducing intrinsic load.
Level 3: Projection	Creative Intelligence	Anticipating how a high power-distance crew member will respond to a challenge; foreseeing communication-breakdown risks.	Requires WM resources to run mental simulations and generate adaptive solutions under time pressure.

**Table 3:** Mapping Triarchic Intelligences onto Maritime ESP Competencies.

Feature	Analytical Intelligence	Creative Intelligence	Practical Intelligence
Definition in maritime context	Cognitive operations for accurate real-time perception, decoding, and evaluation of linguistic and instrumental inputs.	Capacity to generate adaptive communicative and operational solutions when standard procedures fail.	Deployment of domain-specific tacit knowledge to read contextual cues, manage interpersonal dynamics, and calibrate communication.
Key competencies	Precise SMCP comprehension and production; critical interpretation of multimodal navigational data.	Reformulating messages for non-native speakers; improvising when primary equipment fails; constructing novel linguistic formulations.	Calibrating directness to cultural expectations; prioritising competing demands; managing emotional and cognitive state under stress.
Relationship to cognitive load	Generates high intrinsic load in real time; relies on germane load (schema construction) for rapid pattern recognition.	Operates under extreme load when standard schemas fail; depends on working memory freed by practical expertise.	Reduces intrinsic load by chunking through tacit knowledge and schema-driven processing.
Relationship to SA	Level 1 (Perception): accurate decoding of environmental elements and standardised terminology.	Level 3 (Projection): anticipating future states, running mental simulations, improvising adaptive responses.	Level 2 (Comprehension): interpreting pragmatic meaning through contextual knowledge and expert pattern recognition.

**Table 4:** 32-Week Triarchic Maritime ESP Curriculum Architecture.

Weeks	Phase	Module/Theme	Primary Intelligence(s)	Key Instructional Activities	Assessment Moment
1–4	Foundation	Foundations of Maritime English & SMCP	Analytical	SMCP vocabulary acquisition; phonological discrimination drills using VHF recordings; structured listening; written comprehension.	SMCP recognition & production test (formative).
5–8	Foundation	Maritime Instrumental & Navigational Vocabulary	Analytical	Technical-terminology mapping (radar, ECDIS, AIS); reading authentic navigational notices; instrument-display interpretation tasks.	Vocabulary mastery test; instrument-reading comprehension.
9–12	Foundation → Integration	Routine Bridge Communications	Analytical + Practical	Paired SMCP role-plays; structured ship-shore exchanges; pragmatic-norms analysis of authentic VHF corpora.	Oral SMCP performance assessment; pragmatic-appropriacy rubric.
13–16	Integration	Cross-Cultural Communication on the Bridge	Practical	Hofstede-dimension awareness sessions; case studies from Khan et al. (2026); role-plays simulating high power-distance interactions.	Cross-cultural scenario response (written + oral); reflective journal.
17–20	Integration	Crisis Communication: Mayday, Pan-Pan, and Securité	Analytical + Practical	Distress-call structure; recorded analysis of historical maritime emergencies; simulated VHF crisis scripts with VTS role-players.	Simulated distress communication (performance task).
21–22	Integration → Performance	Communication Breakdown and Recovery	Creative + Practical	Equipment-failure improvisation tasks; non-native-speaker comprehension breakdown role-plays; SBAR-style repair sequences.	Breakdown-recovery performance task with debrief.
23–26	Performance	Full-Mission Bridge Simulator Exercises (Routine)	All three (balanced)	Full-bridge simulator runs with multinational student teams; instructor-injected cognitive-load variations; structured post-simulation debriefs.	Simulator performance evaluation; team-level SA rubric.
27–28	Performance	Full-Mission Bridge Simulator (Crisis Scenarios)	Creative (lead) + Analytical + Practical	Simulator scenarios with deteriorating weather, equipment failure, multicultural communication breakdown; recognition-primed decision exercises.	Crisis-management performance task; capstone debrief.
29	Performance	Reflective Practice and Tacit-Knowledge Articulation	Practical	Structured think-aloud reviews of own simulator recordings; expert pilot guest debriefs; cognitive task analysis worksheets.	Reflective portfolio entry; tacit-knowledge articulation interview.
30	Performance	Workplace-Integrated Project	All three (integrative)	Authentic maritime communication artefact analysis (e.g., MAIB or NTSB report); student-led incident reconstruction with linguistic focus.	Project report (written) + oral defence.
31	Performance	Capstone Simulator Integrated Assessment	All three	Integrated full-mission scenario combining navigational, cross-cultural, communicative-breakdown, and crisis-response demands.	Capstone performance assessment (rubric in Table 5).
32	Closure	Reflection, Synthesis, and Transition to Sea Service	Metacognitive	Portfolio review; instructor-mentor feedback session; transition planning to shipboard practice.	Portfolio defence; end-of-course self-assessment.

**Table 5:** Competency Assessment Matrix for Triarchic Maritime ESP.

Intelligence	Target Construct	Recommended Instrument	Curriculum Timing	Validity Considerations
Analytical	Precision of SMCP comprehension and production; instrumental data interpretation; phonological discrimination on degraded audio channels.	Standardised SMCP listening and production test; instrument-reading multiple-choice items; phoneme-discrimination task with VHF noise overlay.	Weeks 4, 8, 12 (formative); week 12 (summative).	High construct validity; relatively unproblematic to design; primary risk is over-reliance leading to wash-back away from creative and practical objectives.
Creative	Adaptive communication when standard SMCP is insufficient; message reformulation; improvised channel-switching.	Performance task: scripted simulator scenario in which standard SMCP fails (e.g., equipment failure + non-native interlocutor); evaluated against an adaptive-communication rubric on five dimensions (recognition of breakdown, reformulation, channel switching, recovery, post-event debrief).	Weeks 21–22 (formative); week 28 (summative).	Lower construct validity than analytical; rubric reliability requires inter-rater training; recommended at least two raters per performance.
Practical	Cross-cultural pragmatic appropriacy; team-level distributed-cognition participation; tacit-knowledge articulation.	Multi-method assessment: cross-cultural scenario response (written + oral); team-level SA observation rubric during simulator runs; tacit-knowledge articulation interview based on simulator recording.	Weeks 16 (formative), 23–26 (formative), 29 (summative).	Lowest construct validity of the three; mitigated by multi-method triangulation; tacit-knowledge interview adapted from cognitive task analysis protocols.

**Table 6:** Cross-Domain Extrapolation Targets and Current Evidentiary Basis.

Domain	Structural Parallels to Maritime	Current Evidentiary Strength	Epistemic Status of Extension
Aviation	ICAO phraseology parallel to SMCP; CRM literature; multicultural cockpits in some contexts; foundational status in Hutchins (1995b).	Strong: extensive CRM and aviation-English research base (Haji Amiri et al., 2025; Helmreich & Merritt, 1998; A. W. Wang & Friginal, 2025).	Theoretically plausible parallel; direct empirical testing of the reweighting model in aviation training contexts recommended.
Emergency Medicine	SBAR-style standardised communication; time-compressed multidisciplinary teams; high consequence asymmetry.	Moderate: protocol-effectiveness research (Shrivastava et al., 2025) and adaptive-expertise scoping reviews (Pelgrim et al., 2023; Hissink et al., 2025) support structural similarity but do not directly test reweighting.	Theoretically plausible hypothesis; empirical reweighting test in emergency-medicine settings recommended.
Military Coalition Operations	Standardised operational vocabulary; high power-distance asymmetries; cross-cultural team dynamics under stress.	Limited: Siegel et al. (2025) scoping review identifies need but does not test reweighting; primary-data evidence sparse.	Hypothesis for future testing; mapping of the model to coalition contexts would require domain-specific operationalisation.
Safety-Critical Industries (Nuclear, Oil & Gas, Chemical)	Standardised operational protocols; multicultural workforces; emergency communication under degraded conditions.	Limited: domain has rich safety-engineering literature but sparse cognitive-pedagogical literature relevant to the reweighting model.	Hypothesis only; empirical work to test even structural parallels recommended before pedagogical extension.

## Data Availability

No new data were created or analyzed in this study. Data sharing is not applicable to this article.
